# The impact of speech type on listening effort and intelligibility for native and non-native listeners

**DOI:** 10.3389/fnins.2023.1235911

**Published:** 2023-09-28

**Authors:** Olympia Simantiraki, Anita E. Wagner, Martin Cooke

**Affiliations:** ^1^Institute of Applied and Computational Mathematics, Foundation for Research & Technology-Hellas, Heraklion, Greece; ^2^Department of Otorhinolaryngology/Head and Neck Surgery, University Medical Center Groningen, University of Groningen, Groningen, Netherlands; ^3^Ikerbasque (Basque Science Foundation), Vitoria-Gasteiz, Spain

**Keywords:** listening effort, cognitive load, speech perception, non-native listeners, pupillometry, growth curve analysis

## Abstract

Listeners are routinely exposed to many different types of speech, including artificially-enhanced and synthetic speech, styles which deviate to a greater or lesser extent from naturally-spoken exemplars. While the impact of differing speech types on intelligibility is well-studied, it is less clear how such types affect cognitive processing demands, and in particular whether those speech forms with the greatest intelligibility in noise have a commensurately lower listening effort. The current study measured intelligibility, self-reported listening effort, and a pupillometry-based measure of cognitive load for four distinct types of speech: (i) plain i.e. natural unmodified speech; (ii) Lombard speech, a naturally-enhanced form which occurs when speaking in the presence of noise; (iii) artificially-enhanced speech which involves spectral shaping and dynamic range compression; and (iv) speech synthesized from text. In the first experiment a cohort of 26 native listeners responded to the four speech types in three levels of speech-shaped noise. In a second experiment, 31 non-native listeners underwent the same procedure at more favorable signal-to-noise ratios, chosen since second language listening in noise has a more detrimental effect on intelligibility than listening in a first language. For both native and non-native listeners, artificially-enhanced speech was the most intelligible and led to the lowest subjective effort ratings, while the reverse was true for synthetic speech. However, pupil data suggested that Lombard speech elicited the lowest processing demands overall. These outcomes indicate that the relationship between intelligibility and cognitive processing demands is not a simple inverse, but is mediated by speech type. The findings of the current study motivate the search for speech modification algorithms that are optimized for both intelligibility and listening effort.

## 1. Introduction

A listener's experience of speech encompasses signals that differ from naturally-spoken forms. For example, mobile devices and domestic voice assistants make extensive use of speech that has been synthesized from text, while speech relayed over public-address systems or in educational settings may have been modified with the goal of improving intelligibility. Increasing effort is being directed at understanding the impact of these different speech types on message reception for native listeners (e.g., Cooke et al., 2013; Rennies et al., 2020). An additional obstacle faces listeners communicating in a second language (L2), for whom non-standard forms of speech might constitute one of the adverse conditions known to negatively-impact non-native listeners (see review by García Lecumberri et al., 2010).

Speech modification algorithms (e.g., Sauert and Vary, 2009; Tang and Cooke, 2012) and numerous studies of speech types and L2 listening focus solely on intelligibility, leaving out other crucial aspects of the speech understanding process (see also Baese-Berk et al., 2023). Although high intelligibility is critical for correct understanding of the intended meaning in a communicative setting, the practical value of intelligibility studies is questionable in real-world scenarios, for a number of reasons. First, in all but the most adverse listening conditions, spoken output technology is already configured to be highly intelligible, and much listening takes place at signal-to-noise ratios (SNRs) which have only a modest impact on the proportion of words likely to be misrecognized (Smeds et al., 2015). Second, even when speech is maximally intelligible, the effort involved for a listener in understanding modified forms of speech may well depend on the how the speech has been altered. This is especially the case for speech generated using synthesis from text (Govender and King, 2018b; Govender et al., 2019). High listening effort has been associated with increased fatigue (Hornsby et al., 2016) and a reduced ability to perform secondary tasks (Gagné et al., 2017). This consideration becomes even more critical for non-native speakers due to their relatively reduced exposure to the second language, which has been shown to impact both speech-in-noise performance (reviewed in García Lecumberri et al., 2010) and listening effort (Borghini and Hazan, 2020). Understanding cognitive processing effort is therefore crucial in designing effective speech enhancement algorithms for listeners across the spectrum of linguistic experience. These considerations motivate the current study, which reports on the outcome of two experiments that attempt to measure the effort involved in speech understanding as a function of speech type, for two groups of listeners that differ in whether they are processing speech in their first or second language.

Proxies for listening effort have been obtained in various ways (see McGarrigle et al., 2014 for a review) such as through the use of questionnaires (Mackersie and Cones, 2011; Dawes et al., 2014), via behavioral metrics such as response times (Houben et al., 2013) or performance on dual tasks (e.g. Wu et al., 2016), and via physiological measures such as heart rate, skin conductance (e.g. Mackersie and Cones, 2011) and pupil dilation (e.g. Zekveld et al., 2010). Pupillometry in particular provides a systematic, objective and continuous measure, with increases in pupil size believed to reflect increased listening effort (Zekveld et al., 2010). Pupil dilation has been shown to increase in challenging speech tasks such as sentence processing in the presence of a competing talker relative to a stationary masker (Koelewijn et al., 2014) and has been used to study the influence of factors such as age and hearing loss when processing speech in noise (Zekveld et al., 2011). The effort required to decode speech has been found to vary as a function of masking noise (Zekveld et al., 2010; Koelewijn et al., 2012, 2014; Zekveld and Kramer, 2014), spectral resolution (Winn et al., 2015), syntactic complexity (Wendt et al., 2016) and attentional focus to spatial location (Koelewijn et al., 2015).

Several previous studies have explored the impact of altered speech types on listening effort. Koch and Janse (2016) conducted an eye-tracking experiment to explore the effect of speech rate on spoken word recognition, using conversational materials with a natural variation in speech rate. While listeners exhibited longer response times for fast speech, there was no speech rate effect on the pupil response. Borghini and Hazan (2020) examined the impact of conversational and clear speaking styles on listening effort in the presence of 8-talker babble noise using an SRT procedure. Apart from the expected finding that listeners tolerated a lower SNR for clear (speech produced to aid understanding) compared to plain (speech produced in quiet) style sentences, both the mean and peak pupil dilations were greater for conversational speech, suggestive of a greater listening effort. The impact of synthetic speech on listening effort has also been investigated, revealing that pupil dilation is sensitive to speech quality. Three studies by Govender and colleagues examined the differences between natural speech and four speech synthesis approaches of differing sophistication, namely Hybrid, Unit Selection, Hidden Markov Model (HMM), and Low-Quality HMM, all drawn from the 2011 Blizzard Challenge (King and Karaiskos, 2011). Govender and King (2018a) tested synthetic speech in noise-free conditions using a dual-task paradigm, finding that synthetic speech led to slower reaction times (suggesting a higher cognitive load) as speech quality decreased. Again using stimuli in quiet, Govender and King (2018b) found greater pupil dilation for synthetic speech compared to its naturally-produced counterpart. Masking noise also led to an increase in pupil dilation for synthetic speech (Govender et al., 2019).

Collectively, findings with forms of speech that differ from canonical “plain” speech highlight a potential to affect a listener's experience, either by reducing effort (clear speech) or increasing effort (synthetic speech). However, it is not clear which factors influence listening effort. One possibility is that effort is related to factors that are somewhat independent of intelligibility, such as naturalness or quality. Synthetic speech, particularly that produced by less sophisticated approaches, sounds clearly unnatural. The finding by Govender and King (2018a) that effort is reduced for more recent state-of-the-art synthesis techniques which are mainly distinguished by their degree of naturalness or quality also supports the idea of a relationship between these factors and increased effort. However, it is also possible that forms of speech (clear or high-quality) that result in a reduction of listening effort are also intrinsically more intelligible than their high effort counterparts such as conversational speech or low-quality synthetic speech. One way to explore these issues is to measure both intelligibility and effort for a range of types of speech, and to determine whether the relationship between the two measures is one of simple inverse (i.e., lower intelligibility tending to result in higher effort), or whether the relationship is more complex, involving such attributes as naturalness and quality.

Consequently, the primary goal of the current study was to determine whether those forms of speech that are well-recognized in noise lead to a reduction in listening effort compared to forms that are more challenging to understand. Four forms of speech were compared: (i) “plain” speech, i.e., an unmodified natural form of speech; (ii) Lombard speech (Lombard, 1911), a naturally-enhanced form of speech resulting from speaking in the presence of noise, known to be substantially more intelligible than plain speech when presented at the same SNR (Dreher and O'Neill, 1957; Pittman and Wiley, 2001; Marcoux et al., 2022); (iii) SSDRC, an algorithmically-modified speech type involving spectral shaping and dynamic range compression, also demonstrated to improve intelligibility (Zorila et al., 2012); and (iv) synthetic speech. These diverse forms were chosen since they are increasingly representative of everyday listening, and, critically, because they exhibit widely-differing intelligibilities when presented in identical amounts of noise (Cooke et al., 2013). For example, in a condition involving speech-shaped masking noise presented at an SNR of –4 dB, Lombard and SSDRC-processed speech showed intelligibility gains of 18 and 29 points respectively relative to plain speech, while synthetic speech had a deficit of nearly 32 points. If high intelligibility has an inverse relationship with processing effort (our first research question), the four speech types of the present study ought to exhibit a very clear ranking of effort given the aforementioned range of intelligibilities.

A second goal of the current study was to examine how different forms of speech impact on effort for non-native listeners. Listening in a non-native language is known to affect intelligibility, with the greatest impact felt under adverse conditions (García Lecumberri et al., 2010; Mattys et al., 2012), broadly defined to include challenging listening conditions such as noise and reverberation and, of relevance for the current study, atypical speech (Cooke and García Lecumberri, 2016). It is an open question as to whether listening effort follows a similar pattern i.e., do non-native listeners also suffer a disproportionate increase in effort when processing challenging forms of speech compared to the effort experienced by native listeners? While non-native speech perception is an important factor in the Framework for Effortful Listening (Pichora-Fuller et al., 2016), there have been relatively few studies of non-native listening effort. Schmidtke (2014) used the visual-world paradigm to explore retrieval effort of English words for both English and Spanish listeners, finding that the latter cohort had a delayed pupil response compared to the former. Borghini and Hazan (2018) compared pupil responses when processing English sentences in quiet and babble noise by native and Italian listeners. When performance was matched across groups, overall mean and peak pupil responses were larger for the Italian group, in both quiet and masked conditions, a finding confirmed in Borghini and Hazan (2020). Peng and Wang (2019) estimated listening effort for native English and two non-native cohorts using a dual-task paradigm in conditions of masking noise and reverberation, revealing slower responses for one non-native cohort. Additionally, non-native listeners reported higher effort in comprehending speech in adverse conditions, measured using the NASA task load index questionnaire (Hart and Staveland, 1988). The current study addresses the issue of whether non-native listening effort follows a similar patterns to that of native listeners in the face of differing speech types.

These research questions are investigated using three metrics—intelligibility, subjective judgements of effort, and pupillary responses—for English sentences produced in four speech types, mixed with speech-shaped noise at three SNRs. In Experiment I (Section 2) the listener cohort consists of native English speakers, while Experiment II (Section 3) involves a group of non-native (Spanish) learners of English.

## 2. Experiment I: native listeners

### 2.1. Methods

#### 2.1.1. Participants

Participants (*N* = 26, 6 males) were young normal-hearing native British English speakers (age range: 18–24, mean = 20.5, SD = 1.8) recruited from among the student population at the University of Edinburgh^1^. Participants were requested not to wear glasses and eye makeup. All had hearing levels better than 25 dB HL in both ears as determined by pure-tone audiometric screening at octave frequencies in the range 125–8,000 Hz. Listeners were paid on completion of the experiment. The study was approved by the Ethics Committee at the University of Edinburgh. Technical problems during recording led to the exclusion of data from two participants.

#### 2.1.2. Speech and masker materials

The Harvard sentence lists (Rothauser et al., 1969) provided the basis for the four distinct speech styles tested in the current study. Harvard sentences typically contain 7–9 words, of which 5 are preselected as keywords for scoring purposes. The speech signals used in the current study were a subset of the speech corpus generated for use in an international challenge of intelligibility-enhancing speech modification algorithms (Cooke et al., 2013).

- In the *plain* condition sentences were produced in quiet conditions by a British English male talker who was asked to speak normally.
- The same talker also produced sentences in the presence of a speech-shaped noise (SSN) masker, resulting in the *Lombard* condition. Lombard speech typically differs from speech produced in quiet. For example, Lombard speech has a flatter spectral tilt, increased fundamental frequency, and some segments exhibit longer durations (Summers et al., 1988).
- Modified speech (*SSDRC* condition) was created by applying the Spectral Shaping and Dynamic Range Compression method (Zorila et al., 2012) to the plain speech sentences. The SSDRC algorithm incorporates ideas from both Lombard and clear speech styles.
- A synthetic speech style (*TTS* condition) was generated using hidden Markov model text-to-speech synthesis. The system employed (Yamagishi et al., 2009) was capable of adapting to individual speakers, so in addition to the (orthographic) sentence text, the TTS system was also provided with additional speech material from the talker who produced the *plain* and *Lombard* sentences.

**Figure 1:**
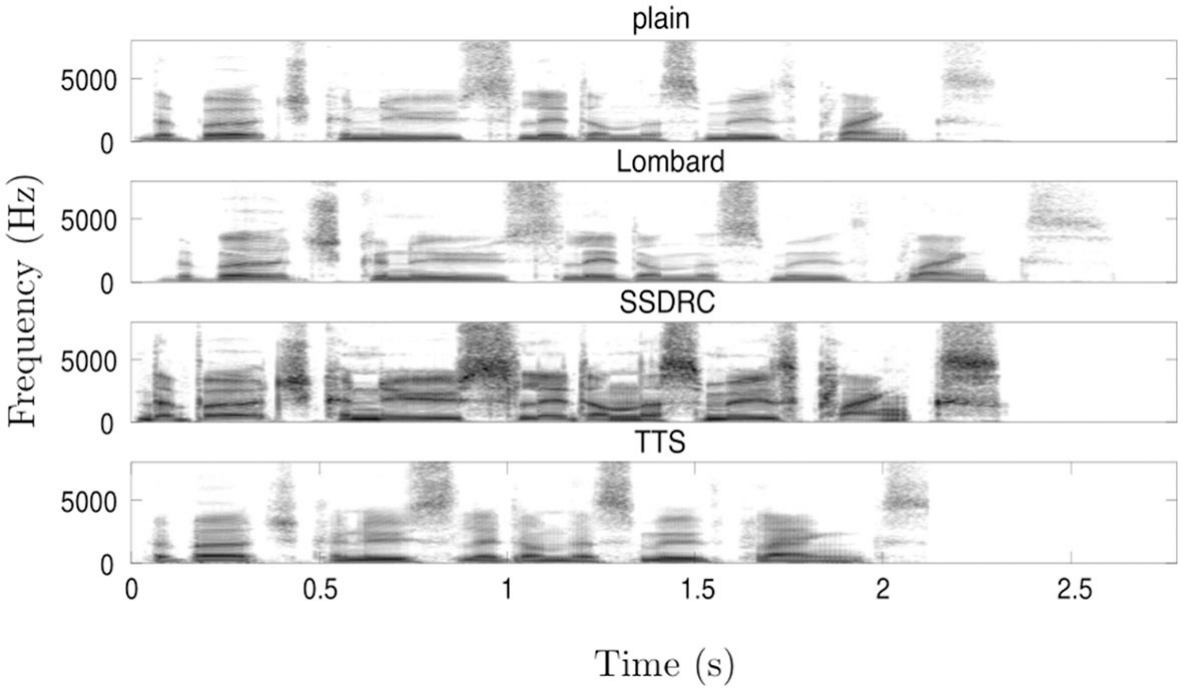
Spectrograms of the phrase “The birch canoe slid on the smooth planks” for each of the speech types tested in the current study.

More details on the corpus from which speech material was drawn are provided in Cooke et al. (2013). Figure 1 shows example spectrograms for the same sentence in each of the four types.

Experimental stimuli were created by mixing sentences in the four styles with a speech-shaped masking noise at each of three SNRs: −1, −3, and −5 dB, resulting in 12 condition blocks. These SNRs were chosen on the basis of recommendations in a study by Ohlenforst et al. (2017b) to avoid values that are too high (low noise) and likely to be effortless for participants, or too low (high noise), potentially leading participants to expend less effort due to the perceived level of difficulty of the task. In pilot tests, the three SNRs chosen produced intelligibility levels both near to and below ceiling. Speech-plus-noise mixtures were created by rescaling the speech signal to achieve the desired SNR in the region where it overlapped with the masker. The resulting mixtures were normalized to have the same root mean square level, and 20 ms half-Hamming ramps applied to the start and end to reduce onset and offset transients.

#### 2.1.3. Procedure

Maskers started two seconds prior to the onset of each sentence, and stopped three seconds after sentence offset. Pupil data from the 1-second interval immediately preceding the onset of the sentence was used for calibration (see Secion 2.1.4).

Listeners heard 12 blocks of stimuli, one block for each combination of speech type and SNR. Each block consisted of 15 target sentences that were used for scoring, preceded by 5 familiarization sentences. None of the 180 (15 × 12) target sentences heard by any given listener were repeated. Block order was balanced across listeners using a Latin square design, and sentence order within blocks was randomized. Before starting the experiment, participants were able to adjust the volume to a comfortable listening level.

The experiment took place in a sound proof studio at the University of Edinburgh. Pupil data was collected using the remote EyeLink 1000 eye-tracker with sampling frequency 500 Hz and the pupil size was measured in terms of pupil area (number of black pixels) while participants listened to sentences through Sennheiser HD-380 pro headphones.

**Figure 2:**
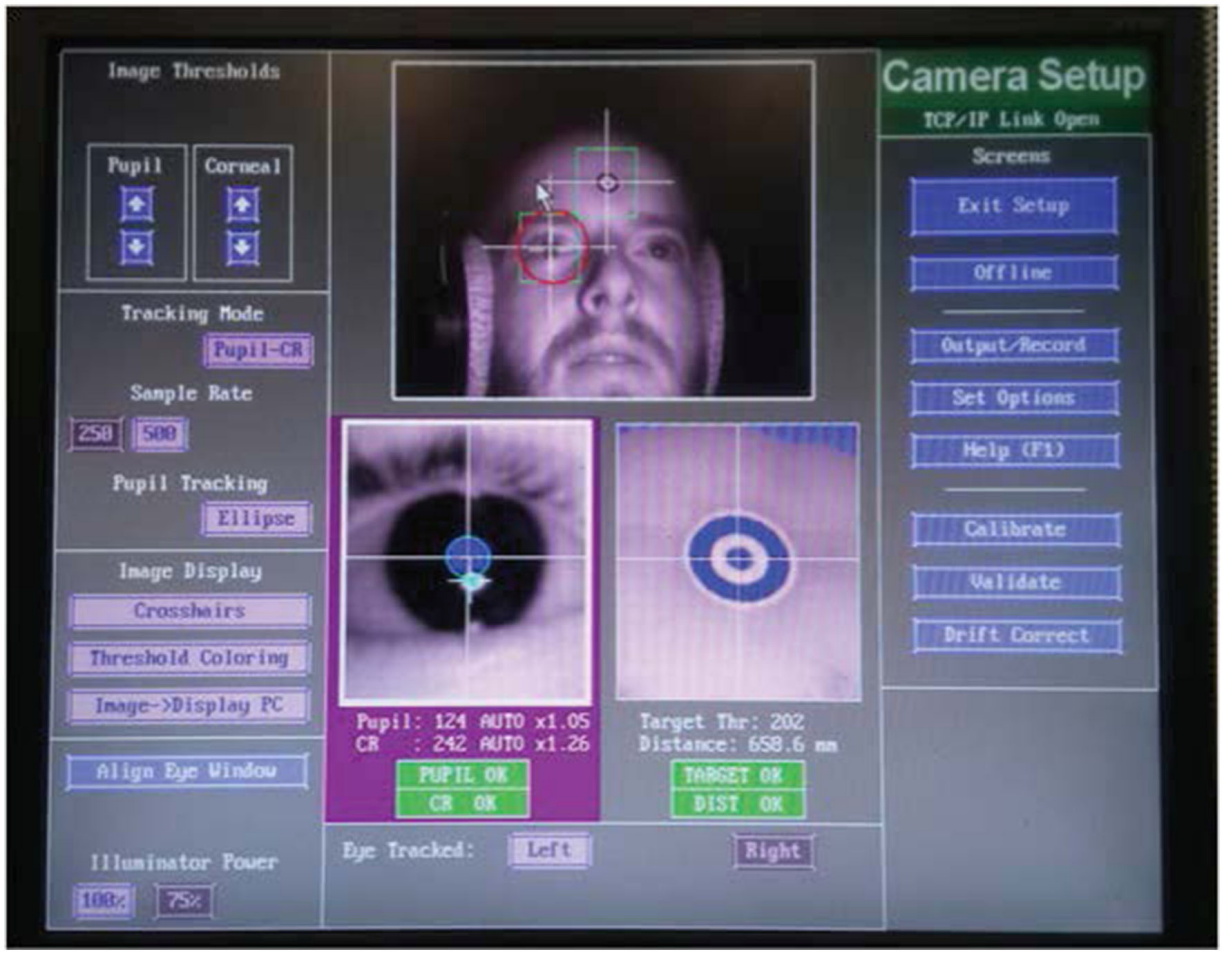
Experimental set up. The image shows a listener during the task through the experimenter's monitor.

**Figure 3:**
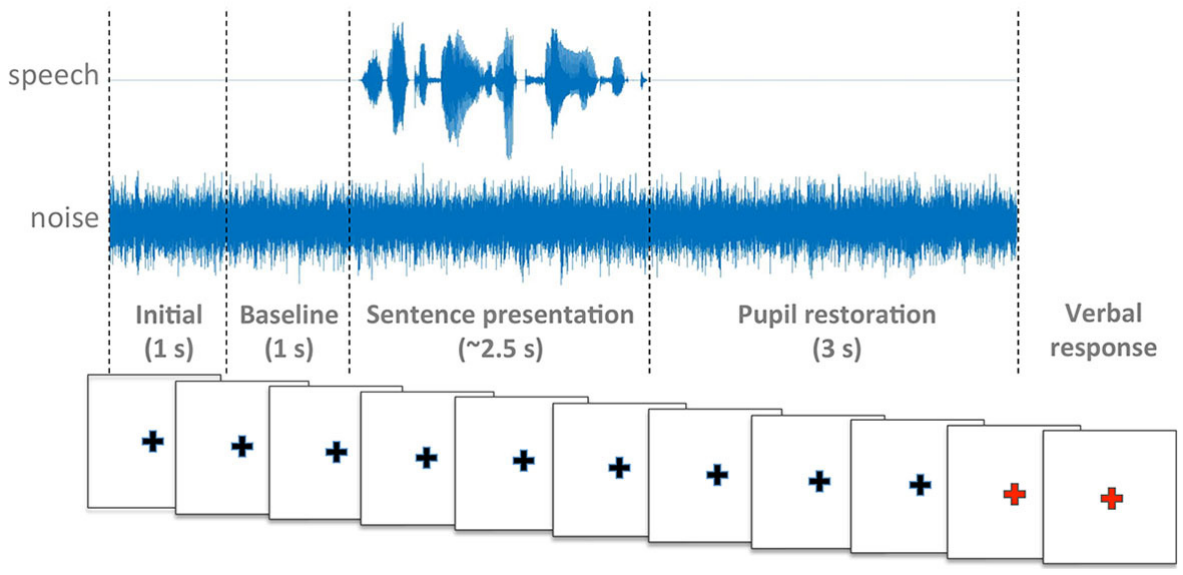
Schematic representation of the experimental procedure.

Participants were seated in front of a computer screen with a white background and a black cross at the center (Figure 2). Participants were instructed to look at the cross while listening to the stimuli. At the end of the trial the cross became red and participants were asked to repeat verbally the words they had heard (Figure 3).

On completion of each block, participants answered the question “How much effort did it take listen to and understand the sentences in this block?” using a numeric rating scale from 0 (no effort) to 10 (very effortful). The experiment was split into two parts of approximately 30 min each, with an intervening 5-min break.

#### 2.1.4. Calibration

**Figure 4:**
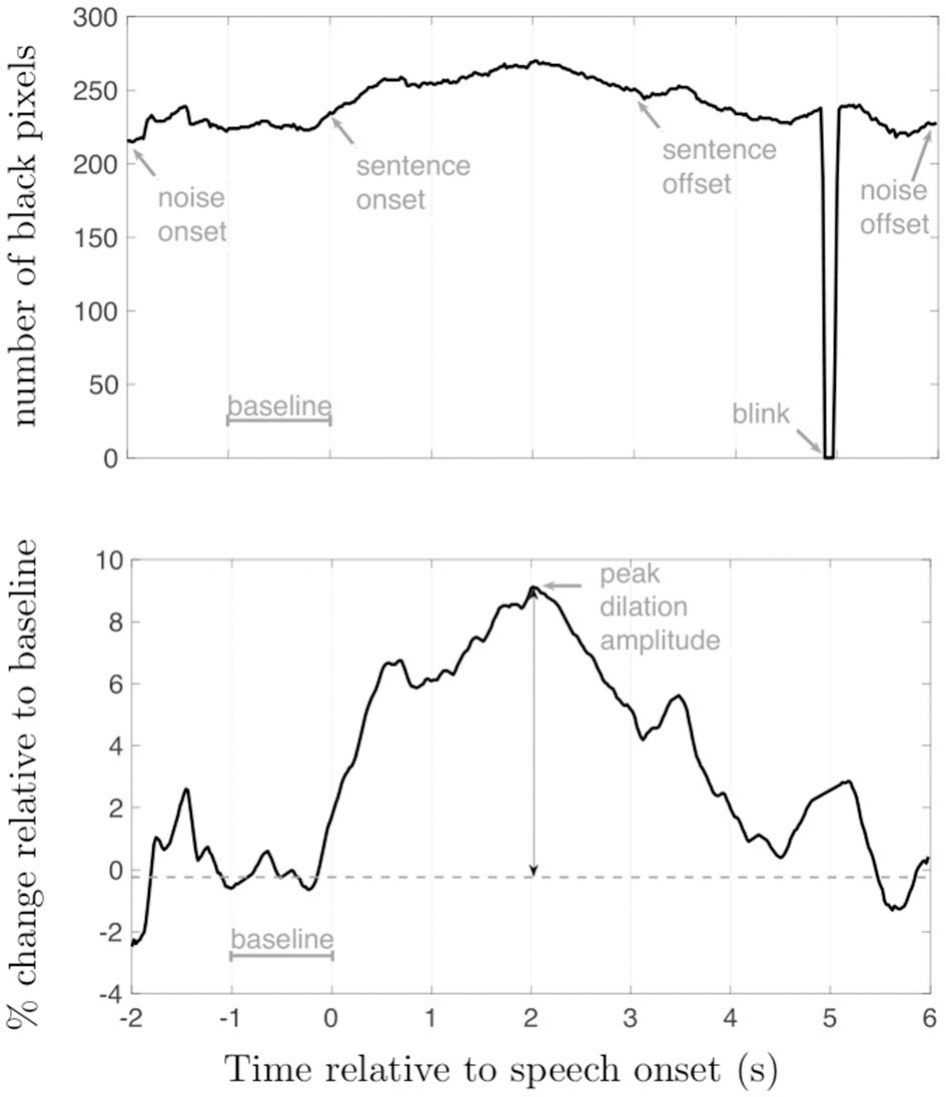
Pupil size variation during a single trial. **(Top)** Uncalibrated pupil area; **(Bottom)** Calibrated pupil diameter. Times are relative to sentence onset at 0 s.

Similar practices to those suggested in Winn et al. (2018) were used in processing raw pupillary responses and in discarding trials. Pupil data from the left eye was used. Pupil area data was first downsampled to 50 Hz and converted to pupil diameter, and the following signal-cleaning procedure (designed, for example, to detect blinks) was applied. For each trial, cases where the pupil size was more than two standard deviations lower than the overall mean pupil size were considered as missing values; these were linearly-interpolated using data in a window that covered the interval from 5 samples prior to the missing value, to 8 samples after the missing value. Following signal-cleaning, event-related pupil dilation (ERPD) was computed from pupil traces following Wagner et al. (2015):


(1) $$\begin{array}{l} \text{ERPD}=\frac{observation-baseline}{baseline}*100 \end{array}$$

where *observation* is the uncalibrated pupil diameter and *baseline* is the mean pupil diameter during the one second interval preceding the onset of the speech. Finally, pupil data were smoothed using a 5-point moving average filter. Figure 4 provides an example of the uncalibrated pupil area (upper plot) and calibrated pupil diameter (lower plot).

#### 2.1.5. Exclusion criteria

Trials with more than 15% missing values, or pupil data with artifacts detected by visual inspection, were excluded from the analysis. Trials for which listeners did not perceive any word correctly were also excluded since pupil dilation is small when intelligibility is close to floor (Zekveld and Kramer, 2014; Ohlenforst et al., 2017b). Overall, these criteria led to the exclusion of 9.7, 15.6, and 30.3 of trials in the −1, −3, and −5 dB SNR conditions respectively (see Supplementary material S1 left table). The high figure for the −5 dB SNR condition is due to fact that on many trials for the TTS speech type no words were identified correctly.

#### 2.1.6. Statistical analyses

Statistical analyses were carried out in *R* [R Core Team (2021) version 3.3.3]. Growth curve analysis (GCA) was used for analyzing the pupil data for each SNR since the entire time-course of the data is taken into account resulting in more meaningful information (Mirman, 2014). A visual inspection of the average pupil dilation responses of all participants and conditions suggested the use of a third-order polynomial for modeling the data within the time window of 0 s (speech onset) until 4.5 s after speech onset. The orthogonal terms of the polynomial can be interpreted as follows: the intercept as the overall mean pupil dilation, the linear term as the overall rate of pupil dilation, the quadratic term as the shape of the peak, and the cubic term as the falling slope of the curve.

Model selection started with the complete model, which included as fixed effects both speech type (*speech_type*) and intelligibility for the three orthogonal terms and as random factors the intercept and the three orthogonal terms per participant. The *lme4* package [Bates et al. (2015) version 1.1-15] was used for fitting a linear mixed-effect model to the data. Intelligibility scores were computed for each sentence with five (the number of keywords in each sentence) as the maximum score. The model fit was evaluated using model comparisons with the *anova* function. Improvements in model fit were evaluated using the log-likelihood ratio, which is distributed as χ^2^ with degrees of freedom equal to the number of parameters added. Statistical significance for individual parameter estimates was assessed using the normal approximation (i.e., treating the *t*-value as a *z*-value). As an outcome of the fitting procedure the intelligibility factor did not improve the model and was removed.

To model the relationship between SNR and speech type with both intelligibility and subjective listening effort ratings, linear mixed-effects analyses were used with SNR and *speech_type* as fixed effects and random per-participant intercepts. *P*-values were obtained using likelihood ratio tests. Intelligibility score percentages were converted to rationalized arcsine units (Studebaker, 1985). *Post-hoc* comparisons used the least-squares means (*emmeans* package; Lenth, 2021), with Tukey adjustment for multiple comparisons. Additionally, repeated-measures correlation between intelligibility scores and subjective listening effort ratings were performed via the *rmcorr* package (Bakdash and Marusich, 2017).

### 2.2. Results

#### 2.2.1. Pupil dilation

**Figure 5:**
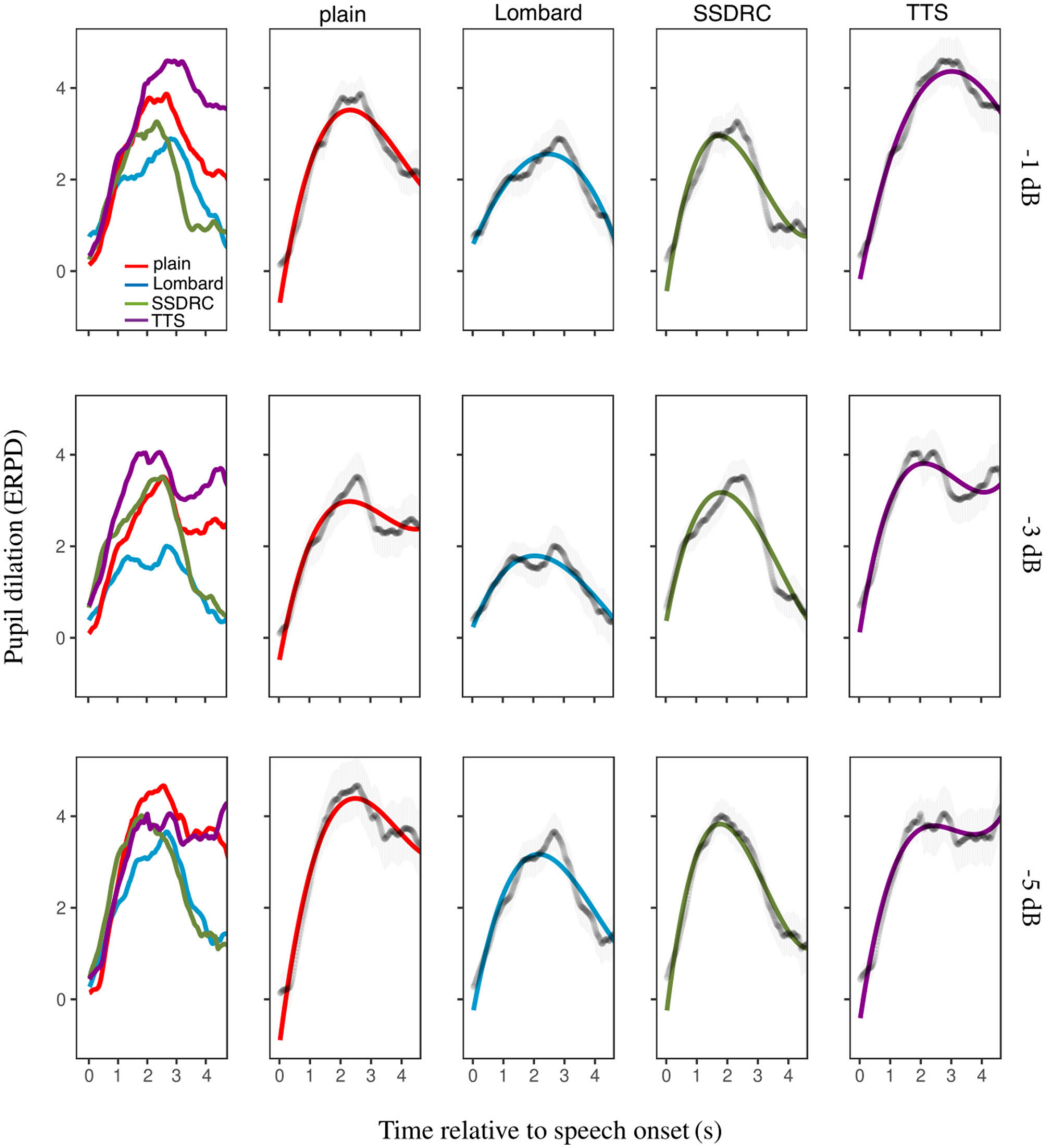
Column 1: mean pupil size over time as a function of speech type. Columns 2–5: the same data plotted for each speech type separately, alongside the fitted model, with error bars denoting ±1 standard error.

Figure 5 (column 1) depicts the ERPD of the calibrated data averaged across participants for each speech style and SNR. For the most favorable SNR, TTS shows the greatest change in pupil dilation over the baseline, followed by plain speech. A similar trend is seen at the intermediate SNR. For the adverse SNR plain speech exhibits the largest relative increase in pupil size. Lombard speech generally results in the lowest ERPD at each noise level.

The best-fitting model was


(2) $$\begin{array}{l} \text{ERPD}\sim \left(t1+t2+t3\right)*speech\text{\_}type+\left(t1+t2+t3|participant\right) \end{array}$$

where *t*1, *t*2, *t*3 represent the 3 orthogonal terms. Columns 2–5 of Figure 5 depict the best-fitted models for each speech type and SNR. At all SNRs, plain speech has a higher overall mean pupil dilation (i.e., the intercept component was greater) and sharper peak (i.e., larger *t*2 apart from in the −5 dB SNR condition) than Lombard speech. For all conditions, the pupil response for SSDRC reaches its peak faster than the remaining speech types (i.e. *t*1 has the greatest absolute value for SSDRC). Finally, overall mean pupil diameter was greatest for the TTS style, except at −1 dB SNR where it was the same as plain speech, and at −5 dB SNR where plain speech had the highest mean pupil diameter followed by TTS. Also, TTS had the flattest overall rate of pupil dilation compared to the Lombard and SSDRC styles. Supplementary Table S2 in the Supplementary material provides estimates of each polynomial term and speech type for the different SNRs, while Supplementary Table S3 shows the interpretation of the GCA results as a function of the orthogonal polynomial terms and SNR.

#### 2.2.2. Intelligibility scores

**Figure 6:**
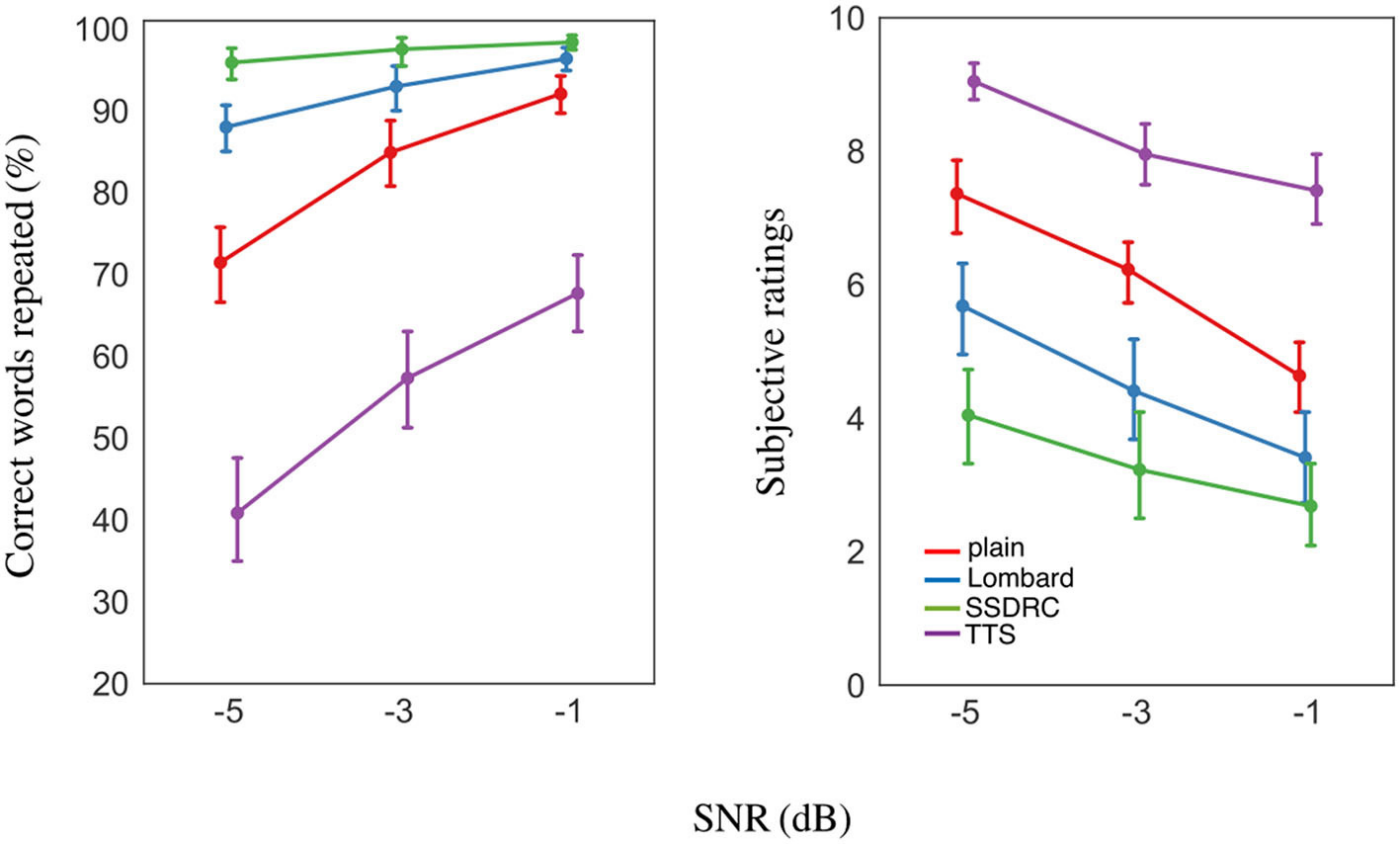
**(Left)** Mean intelligibility scores. **(Right)** Mean subjective listening effort ratings from 0 (no effort) to 10 (very effortful). Error bars in each plot denote ±1 standard error.

The mean percentage of correct words repeated by participants for the different speech types and SNRs is shown in Figure 6 left. Intelligibility is close to ceiling for SSDRC at all SNRs, and for the Lombard style at the most favorable SNR. The ranking of scores was identical at all SNRs viz. from high to low: *SSDRC, Lombard, plain, TTS*.

The mixed-effects analysis showed that intelligibility is linearly related to the interaction of SNR and speech type [*F*_(6, 242)_ = 7.00, *p* < 0.001]. *Post-hoc* pairwise comparisons indicated that intelligibilities for the different speech types differed significantly at each of the 3 SNRs [*p* < 0.01], except for the Lombard/SSDRC pair at –1 dB SNR [*p* = 0.51] and Lombard/plain at the same SNR [*p* = 0.07].

#### 2.2.3. Subjective listening effort ratings

Mean subjective ratings for the different speech types across the 3 SNRs are depicted in Figure 6 right, revealing a clear ranking of speech types that is the inverse of that for intelligibility. Correlation tests verified that intelligibility and subjective ratings were negatively correlated [*r* = −0.87, *p* < 0.001]. Two differences between the outcomes from pupil size and subjective ratings stand out: (i) while listeners rated SSDRC as the least effortful speech style, pupil size was always smallest in the Lombard speech condition, at all SNRs; (ii) synthetic speech produced the largest effort ratings at all SNRs but pupil size for plain speech was larger in the more adverse condition.

A linear mixed-effects model indicated a significant interaction of SNR and speech type [*F*_(6, 242)_ = 1.87, *p* = 0.087]. *Post-hoc* pairwise tests showed that the ratings between the different speech types differed significantly at each SNR [*p* < 0.001] apart from the Lombard/SSDRC pair at −1 dB SNR [*p* = 0.65].

### 2.3. Interim discussion

Experiment I explored the effort (measured both by changes in pupil size and subjective ratings) that native English listeners exert when listening to different speech types in noise, alongside intelligibility measures. The primary research question was whether an inverse relationship exists between intelligibility scores and listening effort. While for subjective effort rating there is a clear inverse relationship with intelligibility, this is not the case for pupillometry-based effort measures. Specifically, Lombard speech had an overall pupil response that was lower than for SSDRC for the two SNRs (–3 and –5 dB) where SSDRC was more intelligible than Lombard speech. Lombard speech and SSDRC were equally intelligible at the least adverse SNR, where differences in pupil dilation responses between the two speech types were not conclusive: all orthogonal terms differed except for overall mean pupil dilation. In line with previous studies, synthetic speech was less intelligible than natural speech (cf. Venkatagiri, 2003; Axmear et al., 2005) and the least intelligible style compared to the plain, Lombard, and SSDRC speech types (cf. Cooke and García Lecumberri, 2016). However, pupillary measures indicated that TTS did not consistently rank as the most effortful across all noisy conditions.

It is possible that the dual processes of spectral shaping (SS) and dynamic range compression (DRC) that underlie SSDRC provide two opportunities to introduce artifacts. SS may have reduced some of the natural variation in the speech signal by smoothing out spectral detail, while DRC modifies the natural amplitude contour of the signal. These processes may have reduced the salience of subtle nuances and variations that are inherent to natural speech, rendering the output more homogeneous and less expressive. It is of interest to note that the one study that examined the quality of speech processed by the SSDRC algorithm (Tang et al., 2018) found its quality to be identical with unprocessed plain speech at a range of SNRs. This outcome is compatible with our finding that subjective ratings bear a simple relationship with intelligibility, but does not illuminate the cause of the less straightforward relationship suggested by the pupillometric outcomes of the current study.

Here, SSDRC had lower *f*_0_, flatter spectral tilt, and shorter duration than Lombard speech (Table 1). Koch and Janse (2016) found that increased speech rate does not have an effect on pupil response in young or older listeners. Thus, the longer duration of Lombard speech may not have contributed to the lower pupil dilation observed for this speech type. However, spectral features of SSDRC such as the greater spectral tilt may have adversely affected listeners. Winn et al. (2015) found that pupil dilation is sensitive to spectral resolution (i.e., the greater the spectral resolution degradation the greater the pupil dilation) even when performance on the intelligibility task was perfect. The closer the speech spectrum is to plain speech the more natural the speech sounds, since the quality of algorithms that boost mid and high frequencies (sacrificing energy below 1,000 Hz) has been judged as poorer compared to alternative approaches (Gabrielsson et al., 1988; Tang et al., 2018). Additionally, in Simantiraki et al. (2020) listeners preferred to enhance speech in noise by increasing spectral tilt as a function of the noise level, and when possible avoided choosing the flattest available tilt. We speculate that modifying the SSDRC algorithm to adapt to the masker's long term spectrum may result in a reduction in the effort required to process this speech form.

**Table 1:** Mean and standard deviation (in parentheses) of duration, spectral tilt and fundamental frequency across the 180 sentences of the experiment.

	**Duration (s)**	**Tilt (dB/octave)**	***f*_0_ (Hz)**
*Plain*	2.06 (0.23)	–5.25 (0.52)	106.50 (4.33)
*Lombard*	2.32 (0.26)	–3.72 (0.56)	138.91 (7.31)
*SSDRC*	2.06 (0.23)	–1.95 (0.58)	111.30 (8.79)
*TTS*	1.95 (0.23)	–5.31 (0.53)	106.21 (1.95)

The plain and TTS styles were rated as more effortful and had greater overall mean pupil dilation responses when compared to Lombard and SSDRC, supporting previous studies that have indicated that listening effort is affected by intelligibility (Zekveld et al., 2010; Zekveld and Kramer, 2014). In addition to the lower intelligibility of TTS, its lower naturalness compared to the other speech types may have contributed to a greater effort required to process this style of speech. This finding echoes that of Govender et al. (2019), who collected naturalness ratings for different speech styles, with listeners reporting lower naturalness for synthetic speech (including hidden Markov model TTS synthesis) compared to natural speech. Unlike the intermediate noise levels, the pupil dilation results showed a different pattern for the most challenging condition, where the intelligibility score for synthetic speech was approximately 40%. In this case, the pupil dilation for plain speech was found to be higher than that of TTS. This may be due to the finding that listening effort is typically maximized for speech-in-noise tasks with intelligibility levels of around 50%, and decreases for conditions with lower intelligibility (Zekveld and Kramer, 2014; Wu et al., 2016; Ohlenforst et al., 2017b).

## 3. Experiment II: non-native listeners

Experiment II explored the listening effort exerted by non-native listeners when processing speech under conditions similar to those of Experiment I (Section 2). Besides the L1 of the listeners, the main difference between the two experiments was in the choice of SNRs, as detailed below. Unless otherwise mentioned, details were the same as in Experiment I.

### 3.1. Methods

#### 3.1.1. Participants

Thirty-one normal-hearing native Spanish listeners (7 males) aged between 18 and 29 (mean age of 20.5, *S*.*D* 2.5 years) took part. Fifteen were monolingual in Spanish and the remaining were bilingual in Spanish and Basque. Listeners were students in the English, German, Translation, and Interpretation Studies Department at the University of the Basque Country, in the second or later year of their studies. Participants reported that they did not suffer from cataracts nor diabetes, and had no known hearing problems. Additionally, they were asked not to wear hard contact lenses or eye makeup during the experiment. Participants underwent a pure tone hearing screening; all had a hearing level less than or equal to 25 dB HL in both ears. Listeners were paid on completion of the experiment. The study was approved by the Ethics Committee at University of the Basque Country.

#### 3.1.2. Speech and masker materials

Experiment II used the same sentence and noise materials as the first experiment, but the SNRs for the non-native cohort were +20, +5, and −1 dB SNR as opposed to −1, −3, and −5 dB, since L2 sentence listening in noise has a more adverse impact than listening in a first language (see review in García Lecumberri et al., 2010). The −1 dB condition was chosen to provide one point of commonality with the native cohort, while the +20 dB condition represents a near noise-free baseline for evaluating the listening effort exerted for the different speech types. The intermediate SNR of +5 dB corresponds to SNRs found in realistic everyday listening scenarios (Pearsons et al., 1977; Smeds et al., 2015; Wu et al., 2018).

#### 3.1.3. Procedure

The experiment took place in a sound-attenuated room at the University of the Basque Country in Vitoria-Gasteiz. Pupil data was collected using a Tobii x3-120 eye-tracker with sampling frequency of 40 Hz. The pupil size was measured in terms of pupil diameter (an estimate of the pupil size in millimeters). Participants listened to sentences through Sennheiser HD-380 pro headphones. The experiment lasted around 1 h and 15 min with a 5 minute break in the middle.

In addition to the task described in Experiment I, listeners scored their level of competence in English for each of speaking, listening, reading and writing, using a scale from 1 (=beginner) to 5 (=native-like), and were also asked to read 10 English sentences which were subsequently used to rate participants' accents. For the rating task, 13 native British English listeners evaluated the accent using an online test (see Supplementary Figure S1). Three evaluators with middle to high contact with Spanish, or that had lived in a Spanish-speaking country for more than a month, were excluded since their rating ability might have been influenced by their exposure to the Spanish language. Evaluators rated two out of the ten sentences (identical for all speakers) on a scale from 1 (=native-like) to 7 (=very accented). Sentences used for rating were “A fresh start will work such wonders” and “The club rented the rink for the fifth night,” both drawn from the Harvard Corpus. The web test lasted approximately 5 min and ratings of 10 evaluators were used for the analysis.

#### 3.1.4. Exclusion criteria

Following identical exclusion criteria as Experiment I. led to the exclusion of 10.4, 4.1, and 1.4% of trials in the −1, +5, and +20 dB SNR conditions, respectively (see Supplementary material S1 right table). Six participants were excluded from subsequent analysis since the above criteria led to <80% of valid trials per participant.

#### 3.1.5. Statistical analyses

Growth curve analysis was used for evaluating pupillary responses. As in the first experiment, third-order polynomials were used to model growth curves and the analysis time window started from the 0 s (speech onset) until 4.5 s after speech onset. Models were constructed by adding as separate fixed effects the speech type, intelligibility, accent ratings (median values), mean reported proficiency level in English (see Supplementary material S6), months lived in a foreign country, and the year of studies. As random effects, the participant id was added. Model fitting showed that only *speech_type* as a fixed factor improved the model and thus the remaining factors were removed from the model. In the +20 dB SNR condition, the interaction between the third orthogonal polynomial term and *speech_type* was removed due to lack of convergence. For the correlation tests, the Pearson correlation coefficient was computed for the non-repeated-measures. Comparisons between accent ratings and intelligibility, year of studies, months that participants have spent in a foreign country, and self-reported mean English level were tested.

### 3.2. Results

#### 3.2.1. Pupil dilation

**Figure 7:**
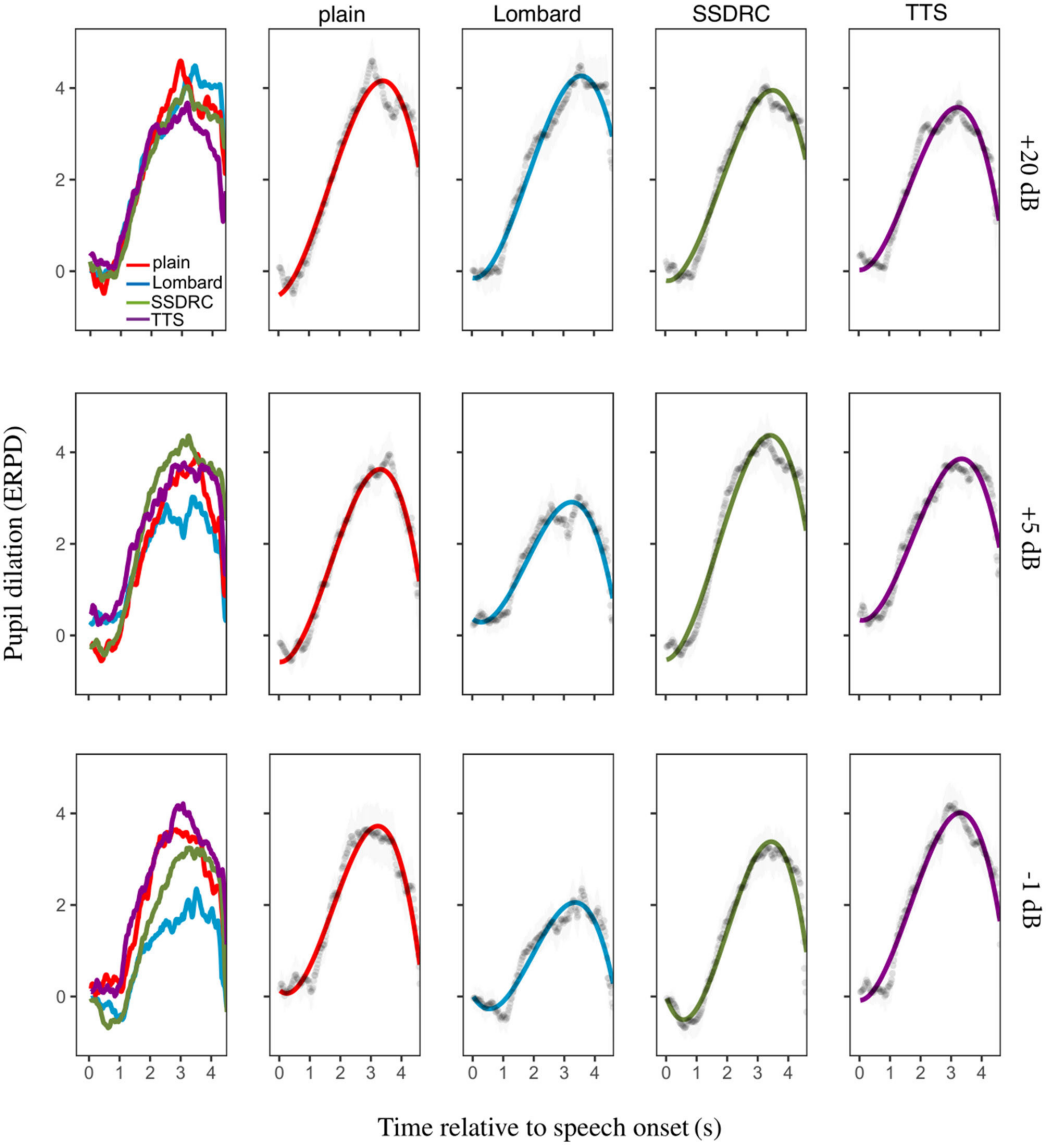
Column 1: mean pupil size over time as a function of speech type. Columns 2–5: the same data plotted for each speech type separately, alongside the fitted model, with error bars indicating ±1 standard error.

Figure 7 (column 1) depicts the ERPD of the calibrated data averaged across-participants for each speech style and SNR. For the least noisy condition, the pupil dilates similarly for all speech types until approximately 2 s when pupil size diverges. A similar but more pronounced pattern of divergence is present for the other noise levels. For the most adverse condition, Lombard speech has the smallest ERPD value, followed by SSDRC, with plain and TTS having the highest ERPDs. The ranking is the same as that manifest by native listeners at the equivalent SNR.

The best-fitting model was identical to that of Experiment I (Equation 2) except for the +20 dB SNR, where the model was the following


(3) $$\begin{array}{l} \text{ERPD }\sim (t1+t2+t3)+speech\_type+t1:speech\_type\\ \;+t2:speech\_type+(t1+t2+t3|participant) \end{array}$$

At +20 dB SNR, plain and Lombard speech had the highest overall mean pupil dilation (as indicated by the intercept component in the model), while SSDRC had lower mean pupil dilation and TTS had the lowest. For both −1 and +5 dB SNR Lombard speech had the lowest mean dilation and the flattest peak dilation compared to the other speech types, except for the +5 dB SNR for which TTS and Lombard had the same peak dilation shape.^2^

#### 3.2.2. Intelligibility scores

**Figure 8:**
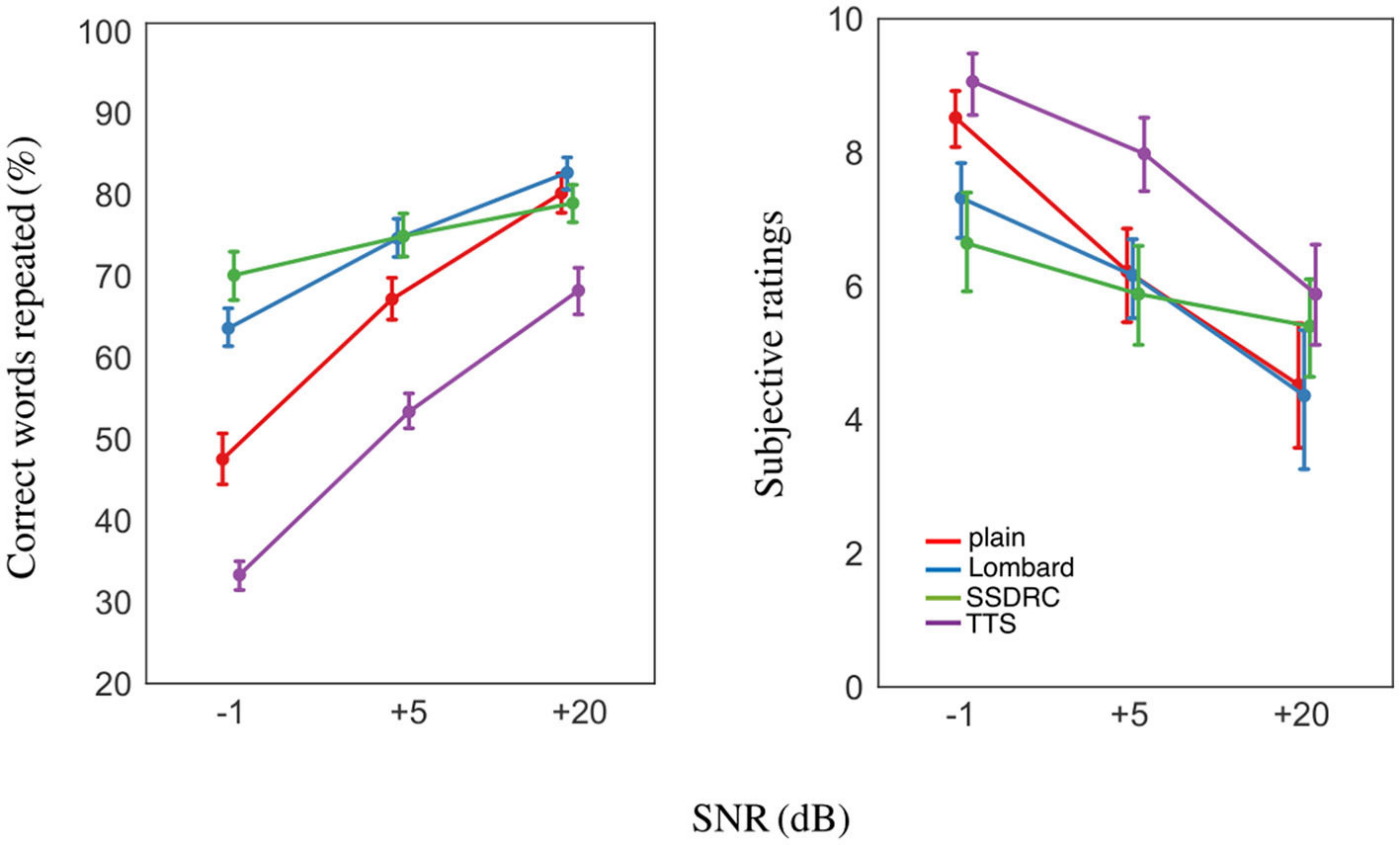
**(Left)** Mean intelligibility scores. **(Right)** Mean subjective listening effort ratings from 0 (no effort) to 10 (very effortful). Error bars denote ±1 standard error.

SSDRC was the most intelligible speech type for all conditions, while TTS was the least intelligible (Figure 8 left). Both natural speech types were less intelligible than SSDRC. However, at the most favorable SNR, plain, Lombard, and SSDRC achieved equal scores. For the condition in common with the native listeners (−1 dB SNR), non-native listeners produced much lower intelligibility scores, with drops of 28, 33, 44 and 34 points for SSDRC, Lombard, plain and TTS. However, the ranking was the same in both experiments.

A linear mixed-effects analysis indicated a significant SNR × speech type interaction [*F*_(6, 275)_ = 17.41, *p* < 0.001]. *Post-hoc* pairwise comparisons suggested that the difference in intelligibility among the speech types was significant at each of the 3 SNRs [*p* < 0.05] except for the Lombard/SSDRC pair at +5 [*p* = 1.00] and +20 dB SNR [*p* = 0.69]. For the most favorable SNR, TTS intelligibility was significantly lower than the other speech types [*p* < 0.001].

#### 3.2.3. Subjective listening effort ratings

Mean subjective ratings for the different speech types across the 3 SNRs are depicted in Figure 8 right. For the adverse noise level, synthetic speech was considered as the most effortful while SSDRC and Lombard speech the least. For the positive SNRs, plain speech was as effortful as SSDRC and Lombard speech, while for the +20 dB SNR all speech types had ratings around the middle of the scale. Subjective effort of all speech types increased with decreasing SNR and was negatively correlated with intelligibility [*r* = −0.71, *p* < 0.001].

A linear mixed model indicated a significant interaction between SNR and speech type [*F*_(6, 275)_ = 5.33, *p* < 0.001]. *Post-hoc* pairwise tests showed that the reported effort for Lombard and SSDRC was similar at each SNR [min *p* = 0.24]. Plain speech was reported as more effortful than SSDRC only for the −1 dB SNR [*p* < 0.001].

Non-native participants rated all speech types as more effortful in the –1 dB condition than their native counterparts in Experiment I, with a near-constant difference of around 3.9 points, except for TTS where the difference was 1.7 points. However, the ranking of speech types was the same for both cohorts.

#### 3.2.4. Accent ratings

Pearson correlations were computed between mean accent ratings for each participant and the number of years of study, months in a foreign country, self-reported mean English level, and intelligibility. Accent ratings were negatively correlated with years of study [*r* = −0.44, *p* < 0.05], months spent in a foreign country [*r* = −0.39, *p* < 0.05] and intelligibility [*r* = −0.41, *p* < 0.05], but not with self-reported English level [*p* = 0.26].

### 3.3. Interim discussion

Experiment II explored intelligibility and the effort exerted by non-native listeners when listening to diverse speech types in noise, and found—as in Experiment I with native listeners—that the relationship between effort and intelligibility is not a simple inverse. In the -1 dB condition common to both experiments, the pattern of pupil responses is similar for native and non-native listeners, and the ranking of intelligibility across speech types is the same for the two groups. Non-native listeners obtained around 35 percentage points lower intelligibility scores compared to natives (Figures 6, 8 left). These findings provide an affirmative answer to our second research question, which asked whether non-native listeners exhibit a similar pattern of listening effort as natives. The observed similarities might be partially explained in terms of intelligibility. Previous studies have shown that the higher the degradation of the speech signal, the larger the decrease in intelligibility and quality, resulting in an increase in pupil dilation (Koelewijn et al., 2012; Zekveld and Kramer, 2014). Scharenborg et al. (2018) also reports that higher degradation of the signal leads to increased lexical competition in both native and non-native listening and increased cognitive effort.

However, as was also the case for Experiment I, the outcomes of Experiment II suggest that factors in addition to intelligibility need to be considered when explaining listening effort. This is most clearly evidenced by the +5 dB condition, where Lombard and SSDRC speech have identical intelligibility for non-natives, but widely-divergent pupil dilations. The finding that effort can vary even when intelligibilities are the same echoes a recent study by Borghini and Hazan (2020) which compared effort for plain speech and clear speech, an enhanced speech type resulting from the instruction to speak clearly. To remove any confounding effect of intelligibility they measured listening effort when intelligibility was equated for native and non-native listeners. Clear speech led to a reduced listening effort when compared to plain speech for both native and non-native listeners in the presence of babble noise.

In Experiment II we also tested a near-clean speech condition, wherein the variations in listening effort among speech types can be considered independently of noise attributes. Our findings in the near-clean condition are somewhat different from the noise-masked conditions in that the naturally-produced speech types appeared to require more effort than the artificial types, as evidenced by pupil dilation responses. It may be that non-native listeners benefit less from acoustic cues that are salient in quiet compared to native listeners, since the typically impoverished non-native linguistic experience might result in less sensitivity to speech modifications. Cooke and García Lecumberri (2012) found that non-native listeners benefitted from Lombard speech in noise but in quiet Lombard speech was less intelligible compared to normal speech. In noise, the acoustic-phonetic changes present in Lombard speech produce a partial release from energetic masking, which benefits native and non-native listeners alike. However, in quiet, these changes may not be beneficial (and might even lead to reduced intelligibility) for non-native listeners compared, for example, to modifications made in foreigner-directed speech. While the latter changes are explicitly intended to assist non-native listeners, some of the modifications in Lombard speech (e.g., durational changes) may interfere with important phonological contrasts, as found by Sankowska et al. (2011).

Finally, although accent ratings (Section 3.2.4) did not have a significant impact on the model used to analyze pupillary responses, our results indicated a negative correlation between accent ratings and intelligibility scores. This finding aligns with a previous study by Borghini and Hazan (2018), which demonstrated that accent ratings were a significant predictor of the estimated SNR level. Specifically, an accent perceived as less native-like was associated with a higher predicted SNR level, indicating poorer performance.

## 4. General discussion

### 4.1. Intelligibility is an imperfect predictor of cognitive effort

The aim of the two experiments reported here was to examine the form of the relationship between listening effort and intelligibility through the use of different types of speech, for both native and non-native listeners. Although intelligibility exhibited a straightforward inverse connection with subjective effort ratings, several findings point to intelligibility being an imperfect predictor of cognitive load as measured by pupil responses. First, despite SSDRC being the most intelligible speech type overall, Lombard speech resulted in the lowest effort. Furthermore, in high noise conditions, the clear disparity in intelligibility between synthetic and plain speech was not reflected in the effort required to process the former. Indeed, in some conditions synthetic speech led to smaller mean pupil dilations than plain speech. The outcomes highlight the complexity of the relationship between intelligibility and listening effort, and suggest that factors beyond mere intelligibility contribute to the cognitive load experienced by listeners in various speech conditions. The nature of these factors remains elusive, but may involve acoustic and phonetic/phonological details that contribute to the perception of naturalness, quality and the presence of artifacts. Additionally, artificial styles such TTS and SSDRC may trigger a sense of unfamiliarity, or mismatch of expectations, and require the deployment of increased attentional resources.

The complexity of the relationship between effort and intelligibility is also supported by prior research investigating the neural mechanisms underlying speech processing. Kim et al. (2021) used naturally spoken words to investigate the impact of noise level variation on cortical speech-in-noise processing, with a focus on regions such as the supramarginal gyrus (SMG) and inferior frontal gyrus (IFG), that are involved in phonological and lexical processing (Hickok and Poeppel, 2007). They indicated that the reduction of the noise level elicited efficient and fast speech processing. Building upon this, Kim et al. (2022) delved into the implications of noise reduction, finding that in the poorest SNR condition, noise reduction significantly increased early SMG activity and decreased late IFG activity, indicating a switch to more immediate lexical access and less effortful cognitive processing, although no improvement in behavioral performance was found.

### 4.2. Subjective ratings of listening effort are not always consistent with physiological measures

In both experiments, there was only a partial correlation between pupillary responses and participants' ratings of effort. The fact that subjective effort ratings were inversely-related to intelligibility strongly suggests that participants were responding based on an introspection of their own performance in the task. A similar outcome was reported by Zekveld and Kramer (2014), whose subjective effort evaluation showed that processing load was higher for lower intelligibility conditions, while subjective ratings and peak pupil dilation were not related to each other, a finding also supported by other studies (Zekveld et al., 2010; Koelewijn et al., 2012). Wendt et al. (2016) concluded that subjective ratings and pupil dilation may well represent different aspects of effort. McGarrigle et al. (2021) demonstrated a within-subject correlation between task-evoked pupil response and the feeling of tiredness caused by listening, but not between pupil response and listening effort. The authors argued that this finding does not necessarily mean that pupil response is not a useful measure of listening effort, as it is possible that the experience of “tiredness” may be easier for participants to report than the experience of “effort.”

### 4.3. Non-native listeners show a tendency for slower pupillary responses compared to native listeners

For all speech types, pupil dilation responses in native listeners occurred within milliseconds of sentence onset, while for non-native listeners they occurred approximately one second later (Figures 5, 7). The slower rise of pupil size might signify greater cognitive demands (Van Der Meer et al., 2010). This observation aligns with earlier research indicating that non-native listeners invest more cognitive effort compared to their native counterparts. Pupillometry results in Borghini and Hazan (2018, 2020) indicated that listening effort, as measured by mean and peak dilation, is higher for non-native listeners than for native listeners during sentence identification, even when intelligibility is matched. Further insights from Weber and Cutler (2004) revealed longer fixation on distractor images for non-native listeners, as well as observations in Schmidtke (2014) of a delayed pupil response in bilingual speakers within a visual-world paradigm, indicative of increased retrieval effort. Additionally, Song and Iverson (2018) employed electroencephalography and detected greater neural tracking of target speech among non-native listeners, despite achieving lower intelligibility scores. This heightened neural engagement was attributed to the increased challenges non-native listeners face in speech recognition, which may result in heightened attention to the speech signal. Other studies have compared reaction times of native and non-native listeners. Lam et al. (2018) explored listening effort through response times within a single-task paradigm. They found that even in conditions of perfect intelligibility, non-native listeners required longer response times compared to native listeners, indicating longer processing times for the former group. In another study by Visentin et al. (2019), examination of response times underscored that when the target signal is masked by fluctuating noise, non-native listeners require extended processing time compared to their native counterparts. One factor that might contribute to increased effort for non-native listeners is the presence of competing words triggered by their first language.

### 4.4. Limitations

One limitation of this study is that the two experiments were conducted in different settings using different eyetrackers, allowing for indirect comparisons only. The primary distinction between the eye trackers lies in their sampling frequencies: while the EyeLink 1,000 operates at 500 Hz, the Tobii x3-120 samples at 40 Hz. However, downsampling to 40 Hz should not significantly impact data quality and the ability to capture pupil dilation information. The pupil size changes slowly, so a sampling frequency even of 30 Hz is sufficient (Winn et al., 2018). Our findings are based on ERPD, a transformation representing the percentage of pupil size deviation from the baseline, which should remain unaffected by the variance in eye trackers. Another limitation is that Experiment I lacked a quiet condition which prevented us from evaluating the impact of speech naturalness in a noise-free environment for native listeners. Finally, the present study involved a stationary masker, which is not representative of many real-world adversities. A natural extension would be to explore the impact of informational masking using speech from one or more talkers as the masker. An intriguing possibility is that the less natural the target speech type, the easier it is for listeners to distinguish target from background speech. In such a scenario, it may be that Lombard speech will exhibit greater processing demands than SSDRC.

### 4.5. Potential impact

Our findings underscore the importance of optimizing noise reduction algorithms to modify speech with the joint goals of improving intelligibility and reducing listening effort. Noise-reduction algorithms, such as those used in hearing aids, do not necessarily lead to a boost in intelligibility, as they are designed to attenuate background noise, albeit often at the expense of “speech naturalness” (e.g., Bentler et al., 2008). Nevertheless, NR can effectively alleviate listening effort (e.g., Ohlenforst et al., 2017a). As noted by Kim et al. (2022), even at a “mild” level of NR strength—when the speech does not sound too artificial – listeners can benefit from enhanced cortical speech-processing effects. By adopting insights from Lombard speech, identified overall as the least effortful speech type in our experiments, speech enhancements could increase intelligibility and mitigate listening effort. This may reduce listener fatigue, maintain the capacity for concurrent secondary tasks, and increase a listener's overall experience.

## 5. Conclusions

This paper presents the results of two experiments examining the impact of speech type on intelligibility and listener effort for native and non-native listeners. Four different speech types (plain, Lombard, artificially enhanced, and synthetic speech) were tested in the presence of speech-shaped noise. Despite not always being the most intelligible form, Lombard speech generally required the least listening effort for both native and non-native listeners, while synthetic speech required higher effort demands for native listeners, particularly at intermediate noise levels. At a common SNR, native and non-native listeners exhibited similar effort patterns across the speech types. These results indicate that intelligibility and listening effort are not merely inversely related, but that other characteristics inherent to each speech type play a significant role. Further research is needed to better understand which features of speech lead to greater or less listening effort.

## Data availability statement

The raw data supporting the conclusions of this article will be made available by the authors, without undue reservation.

## Ethics statement

The studies involving humans were approved by the Informatics Ethics Panel, affiliated with the School of Informatics at the University of Edinburgh (first experiment) and by the Comité de Ética para las Investigaciones con Seres Humanos (CEISH-UPV/EHU), affiliated with the Universidad del País Vasco/Euskal Herriko Unibertsitatea (UPV/EHU) (second experiment). The studies were conducted in accordance with the local legislation and institutional requirements. The participants provided their written informed consent to participate in this study. Written informed consent was obtained from the individual(s) for the publication of any potentially identifiable images or data included in this article.

## Author contributions

OS assumed primary responsibility for executing the study, including data collection, and analysis. OS took the lead in writing the initial draft of the manuscript, which was critically reviewed by MC and AW. All authors collaborated in the conceptualization and design of the study and contributed to the interpretation of the results.

## Footnotes

^1^This experiment was carried out during a placement by the first author at the University of Edinburgh as part of the ENRICH project.

^2^See Supplementary material for more details. Supplementary Table S4 lists estimates of each polynomial term while Supplementary Table S5 provides an interpretation of growth curves.
