# Supplementary material for: The impact of speech type on listening effort and intelligibility for native and non-native listeners
Source: Front Neurosci. 2023 Sep 28;17:1235911. doi: 10.3389/fnins.2023.1235911 (PMC10568627; doi:10.3389/fnins.2023.1235911)
Supplement: Supplementary file 1 [file Data_Sheet_1.PDF]

Supplementary Material

0.1 Trial exclusions

		Speech type			
		SSDRC	plain	TTS	Lombard
SNR	-5	0.3%	6.1%	23.3%	0.6%
	-3	0.3%	1.7%	13.3%	0.3%
	-1	0%	0.8%	8.3%	0.6%

		Speech type			
		SSDRC	plain	TTS	Lombard
SNR	-1	0.5%	3.1%	5.6%	1.2%
	$+5$	0.3%	1.3%	2.3%	0.2%
	$+20$	0.1%	0.1%	1.1%	0.1%

Table S1. The proportion of trials that were removed from the analysis for native (left table) and non-native (right table) listeners.

0.2 Growth curve analysis

0.2.1 Experiment I: native listeners

Speech type	-1	-3	-5
Intercept:plain	2.45 (0.30)	2.35 (0.36)	3.03 (0.41)
Intercept:Lombard	-0.97 (0.04)*	-1.20 (0.04)*	-0.91 (0.04)*
Intercept:SSDRC	-0.84 (0.04)*	-0.60 (0.04)*	-0.83 (0.05)*
Intercept:TTS	0.81 (0.04)*	0.80 (0.04)*	-0.22 (0.04)*
t1:plain	4.74 (2.63)	6.95 (2.67)	10.16 (3.57)
t1:Lombard	-6.47 (0.61)*	-9.67 (0.63)*	-10.41 (0.68)*
t1:SSDRC	-9.20 (0.59)*	-14.36 (0.66)*	-14.17 (0.70)*
t1:TTS	8.06 (0.59)	0.11 (0.62)	0.56 (0.70)
t2:plain	-14.05 (2.61)	-8.82 (2.01)	-13.53 (2.76)
t2:Lombard	5.35 (0.61)*	2.11 (0.63)*	0.98 (0.68)
t2:SSDRC	3.82 (0.59)*	-3.11 (0.66)*	0.71 (0.70)
t2:TTS	0.83 (0.59)	0.12 (0.62)	8.09 (0.70)*
t3:plain	4.56 (1.53)	4.03 (1.26)	4.79 (1.86)
t3:Lombard	-6.43 (0.60)*	-2.33 (0.63)*	-1.17 (0.68)
t3:SSDRC	1.79 (0.59)*	0.18 (0.66)	1.74 (0.70)*
t3:TTS	-3.02 (0.59)*	2.22 (0.62)*	1.66 (0.70)*

Table S2. Summary of estimates of intercept and orthogonal polynomial terms ($t1$, $t2$, $t3$) with plain speech as baseline for the different SNRs. The standard error is shown in parentheses and the asterisk indicates those conditions significantly different from baseline.

Term	Interpretation	Order	-1	-3	-5
Intercept	overall mean pupil dilation	greater to lower	TTS = plain ≠ SSDRC = Lombard	TTS ≠ plain ≠ SSDRC ≠ Lombard	plain ≠ TTS ≠ SSDRC ≠ Lombard
Linear	overall pupil dilation rate	steeper to flatter	SSDRC ≠ Lombard ≠ plain ≠ TTS	SSDRC ≠ Lombard ≠ plain = TTS	SSDRC ≠ Lombard ≠ plain = TTS
Quadratic	shape of peak (height and width of the curve)	sharper to flatter	TTS = plain ≠ SSDRC ≠ Lombard	SSDRC ≠ plain = TTS ≠ Lombard	plain = SSDRC = Lombard ≠ TTS
Cubic	falling slope	faster to slower	Lombard ≠ TTS ≠ plain ≠ SSDRC	Lombard ≠ plain = SSDRC ≠ TTS	Lombard = plain ≠ TTS = SSDRC

Table S3. Interpretation of each orthogonal polynomial term and results as a function of SNR. Results are ordered based on the 3rd column. The symbol '=' signifies that the speech types were not statistically significantly different and '≠' the opposite.

0.2.2 Experiment II: non-native listeners

Speech type	+20	+5	-1
Intercept:plain	2.34 (0.56)	1.89 (0.47)	2.11 (0.42)
Intercept:Lombard	0.07 (0.04)	-0.17 (0.04)*	-1.17 (0.05)*
Intercept:SSDRC	-0.12 (0.04)*	0.50 (0.04)*	-0.58 (0.05)*
Intercept:TTS	-0.25 (0.05)*	0.44 (0.04)*	0.24 (0.05)*
t1:plain	18.61 (3.40)	15.94 (3.32)	12.82 (2.95)
t1:Lombard	1.09 (0.60)	-6.62 (0.57)*	-4.18 (0.62)*
t1:SSDRC	-0.58 (0.60)	3.87 (0.57)*	3.31 (0.62)*
t1:TTS	-6.29 (0.60)*	-2.06 (0.58)*	2.33 (0.66)*
t2:plain	-9.01 (1.81)	-9.77 (1.56)	-10.27 (1.56)
t2:Lombard	2.70 (0.60)*	2.77 (0.57)*	5.23 (0.62)*
t2:SSDRC	2.24 (0.60)*	0.44 (0.57)	3.71 (0.62)*
t2:TTS	-0.27 (0.60)	2.39 (0.58)*	1.00 (0.66)
t3:plain		-7.21 (0.92)	-7.09 (1.09)
t3:Lombard		2.65 (0.57)*	1.83 (0.62)*
t3:SSDRC		1.81 (0.57)*	-1.09 (0.62)
t3:TTS		2.38 (0.58)*	1.38 (0.66)*

Table S4. Summary of estimates of intercept and orthogonal polynomial terms ($t1$, $t2$, $t3$) with plain speech as baseline for the different SNRs. The standard error is shown in parentheses and the asterisk indicates the significant different conditions from baseline.

Term	Interpretation	Order	+20	+5	-1
Intercept	overall mean pupil dilation	greater to lower	Lombard = plain $\neq$ SSDRC $\neq$ TTS	SSDRC $\neq$ TTS $\neq$ plain $\neq$ Lombard	TTS $\neq$ plain $\neq$ SSDRC $\neq$ Lombard
Linear	overall pupil dilation rate	steeper to flatter	TTS $\neq$ plain = Lombard = SSDRC	Lombard $\neq$ TTS $\neq$ plain $\neq$ SSDRC	Lombard $\neq$ plain $\neq$ SSDRC = TTS
Quadratic	shape of peak (height and width of the curve)	sharper to flatter	plain = TTS $\neq$ Lombard $\neq$ SSDRC	SSDRC = plain $\neq$ Lombard = TTS	plain = TTS $\neq$ SSDRC $\neq$ Lombard
Cubic	falling slope	faster to slower		plain $\neq$ Lombard = SSDRC = TTS	SSDRC = plain $\neq$ Lombard = TTS

Table S5. Interpretation of each orthogonal polynomial term and results as a function of SNR. Results are ordered based on the 3rd column. The symbol '=' signifies that the speech types were not statistically significant different and '$\neq$' the opposite.

0.3 Accent evaluation - web test

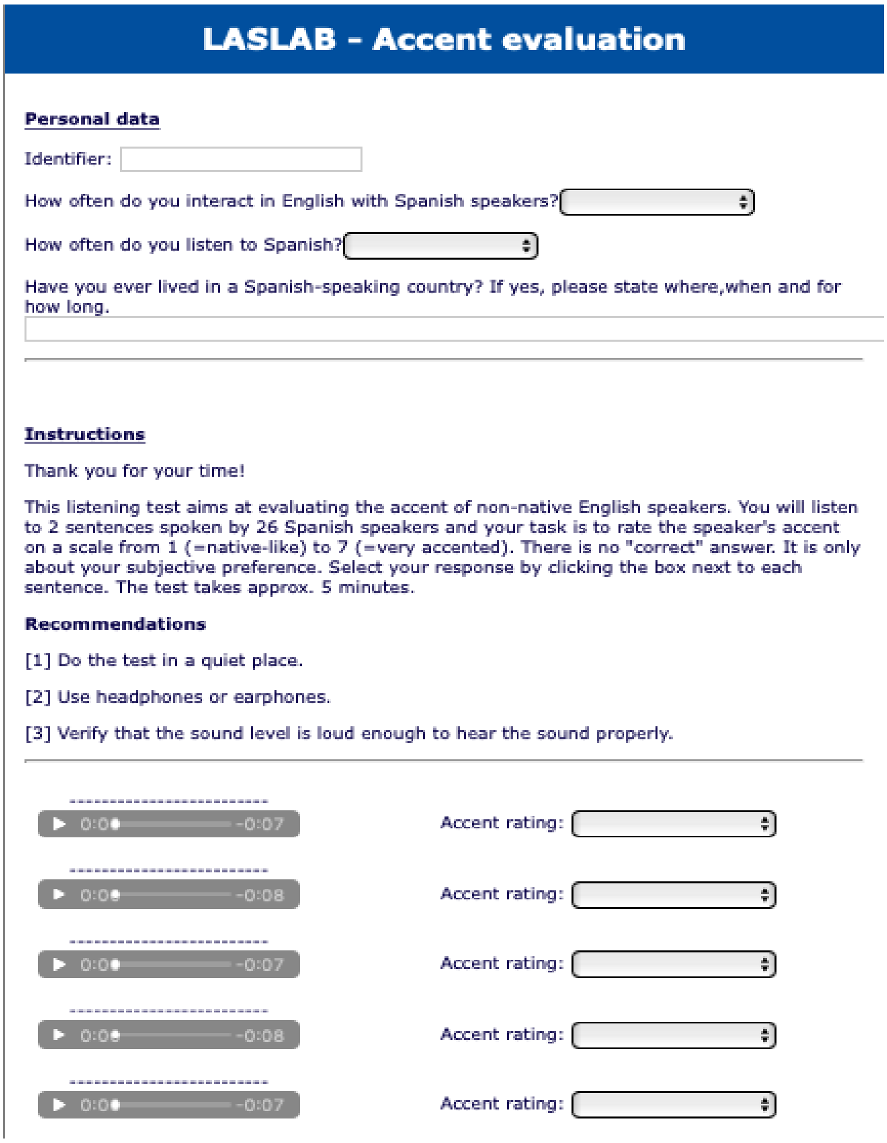

Figure S1. Online test with which native British English listeners evaluated the accent of the non-native listeners.

0.4 Reported proficiency level in English

Participant id	Speaking	Listening	Reading	Writing
**5_1**	4	4	5	4
**5_2**	4	3	3	4
**5_3**	3	4	4	4
**5_4**	3	4	4	4
**5_5**	4	4	4	4
**5_6**	4	4	4	4
**5_7**	5	4	5	5
**5_8**	3	3	4	4
**5_10**	4	4	4	4
**5_11**	4	4	4	4
**5_12**	3	4	4	4
**5_15**	5	5	5	5
**5_16**	4	4	4	4
**5_17**	4	3	4	3
**5_19**	4	4	4	4
**5_20**	3	3	3	3
**5_22**	3	4	4	3
**5_24**	3	3	4	3
**5_25**	4	4	4	4
**5_26**	4	4	4	4
**5_27**	4	3	4	3
**5_28**	4	4	4	4
**5_29**	4	4	3	3
**5_30**	3	4	4	4
**5_31**	4	4	4	4
**mean(SD)**	3.76(0.58)	3.80(0.49)	4.00(0.49)	3.84(0.54)

Table S6. Reported proficiency level in English of the participants included in the analysis.
